# Differential effects of elevated nest temperature and parasitism on the gut microbiota of wild avian hosts

**DOI:** 10.1186/s42523-021-00130-3

**Published:** 2021-10-02

**Authors:** Melissa R. Ingala, Lauren Albert, Alyssa Addesso, Mackenzie J. Watkins, Sarah A. Knutie

**Affiliations:** 1 Department of Vertebrate Zoology, National Museum of Natural History, Washington, D.C. USA; 2 Department of Ecology and Evolutionary Biology, University of Connecticut, Storrs, CT USA; 3 Institute for Systems Genomics, University of Connecticut, Storrs, CT USA

**Keywords:** Birds, Climate change, Gut microbiota, Nest parasites, Temperature

## Abstract

**Background:**

Changes in wild animal gut microbiotas may influence host health and fitness. While many studies have shown correlations between gut microbiota structure and external factors, few studies demonstrate causal links between environmental variables and microbiota shifts. Here, we use a fully factorial experiment to test the effects of elevated ambient temperature and natural nest parasitism by nest flies (*Protocalliphora sialia*) on the gut microbiotas of two species of wild birds, the eastern bluebird (*Sialia sialis*) and the tree swallow (*Tachycineta bicolor*).

**Results:**

We find that bacterial communities from the nestlings of each host species show idiosyncratic responses to both heat and parasitism, with gut microbiotas of eastern bluebirds more disrupted by heat and parasitism than those of tree swallows. Thus, we find that eastern bluebirds are unable to maintain stable associations with their gut bacteria in the face of both elevated temperature and parasitism. In contrast, tree swallow gut microbiotas are not significantly impacted by either heat or nest parasitism.

**Conclusions:**

Our results suggest that excess heat (e.g., as a result of climate change) may destabilize natural host-parasite-microbiota systems, with the potential to affect host fitness and survival in the Anthropocene.

**Supplementary Information:**

The online version contains supplementary material available at 10.1186/s42523-021-00130-3.

## Background

Host-associated gut microbiota are critical for host functioning and fulfill key roles such as provisioning nutrients for the host [1, 2], priming the host immune system, [3, 4] and detoxifying xenobiotic compounds [5–7]. Over the past two decades, our knowledge of microbiota patterning as it relates to host evolution (e.g., [8, 9]) and ecology (e.g., [10, 11]) has increased. While these studies are useful for identifying important correlations between host intrinsic factors and gut community structure, few studies demonstrate causal links between external forces and changes in the gut microbiota.

Understanding how and why gut bacterial communities change in response to specific factors requires careful experimental manipulation. In many cases, such causal links have been inferred through the use of germ-free laboratory models that allow for the controlled testing of effector variables. For example, the inoculation of microbiota characteristic of patients suffering from rheumatoid arthritis was shown to produce the disease phenotype in germ-free mice [12]. Studies addressing causal factors influencing the gut microbiota in non-model animals are rare. Experimental studies performed on free-ranging wild hosts are even rarer despite a growing body of research suggesting that animal-associated microbiotas may inform wildlife health and conservation [13–15].

An important variable with the potential to destabilize host-associated microbial communities is ambient temperature. Given predictions that rising global temperatures will lead to substantial biodiversity loss (e.g., [16–18]), it is reasonable to question how a warming climate might impact or disrupt associations between wildlife hosts and their symbiotic bacterial communities. To date, most studies examining the effects of ambient temperature on microbiota memberships have been performed on ectothermic animals, such as oysters [19], ticks [20], and corals [21]. Fewer studies have tested the effects of ambient temperature on the microbiota of endothermic homeotherms, such as mammals and birds (but see [22]). Furthermore, many field microbiome studies sample animals across different seasons and geographic sites, which may correlate with ambient temperature but may also be confounded by unmeasured variables associated with seasonality, such as variation in rainfall (e.g. [23, 24]). Field-based microbiome studies also rarely investigate the integration of temperature with other environmental stressors, including natural parasitism, the presence of environmental toxins, or habitat loss [25].

**Fig. 1:**
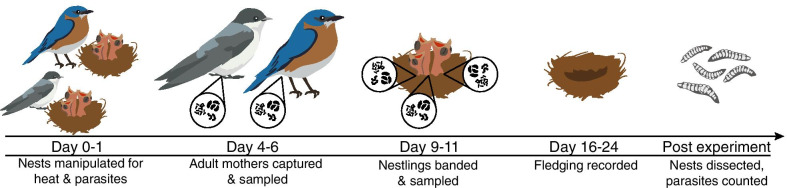
Eastern bluebird and tree swallow nests were manipulated to test the effects of heat treatment and parasitism on microbiota. Days represent nestling age from hatch date

To address these knowledge gaps, we performed a fully-crossed field experiment to test the effects of elevated nest temperature and natural parasitism on the microbiotas of two free-ranging wild bird species, the eastern bluebird (*Sialia sialis*) and the tree swallow (*Tachycineta bicolor*) (Fig. 1). Unlike previous studies, our use of experimental heat manipulation during a single summer relieves the effect of confounding variables introduced by studying temperature changes over seasons. Both eastern bluebirds and tree swallows are box-nesting birds, making them a tractable and easily manipulatable wild system. In addition, both bird species are parasitized by the same species of blowfly, *Protocalliphora sialia*. This parasite does not show an obvious preference for either host species, and previous studies have shown that differences in parasite density between the species are due to differences in host tolerance and resistance [26, 27]. Adult *P. sialia* are non-parasitic but lay their eggs in the nests of birds soon after nestlings hatch. The larvae then feed externally on the blood of nestlings [28]. Larvae of *P. sialia* can be experimentally removed using insecticidal sprays that do not negatively impact bird nestling survival [29–31]. In a related study, the interaction of elevated nest temperature and parasitism was shown to have physiological effects on eastern bluebirds and tree swallows. Bluebird nestlings, but not tree swallows, suffer higher parasite loads and lower body mass when exposed to heat and parasites [32]. This data suggests that host physiology may mediate the interaction between heat and parasitism, leading to different outcomes between host species. Because previous studies show that the early life microbiota may play a key role in mediating later parasitism outcomes (e.g., [33, 34]), it is possible that the microbiota may mediate the effects of heat and nest parasitism, probably via the immune system. We therefore tested whether heat and parasitism could cause a shift in the gut microbiota of eastern bluebirds and tree swallows. We hypothesize that tree swallows, which are generally more tolerant and resistant to both nest parasites and heat, should maintain their gut microbiota structure, while the more susceptible eastern bluebirds should show destabilized microbiotas.

## Results

### Composition and diversity of eastern bluebird and tree swallow microbiota

**Fig. 2:**
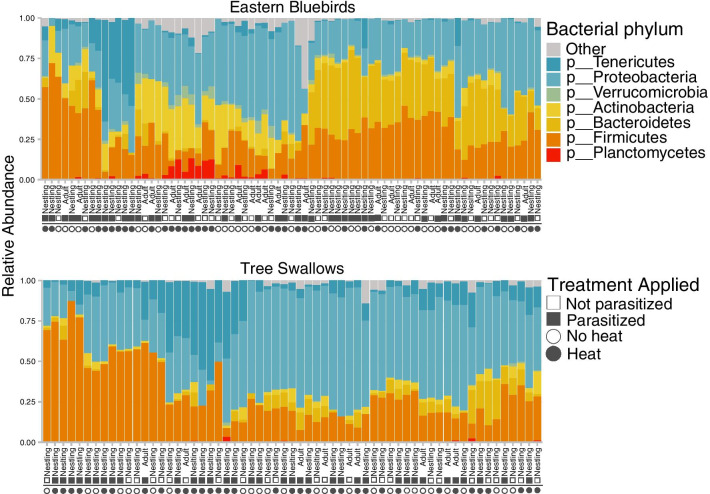
Proportional abundance of bacterial phyla identified from eastern bluebird (top) and tree swallow (bottom) microbiota arranged by hierarchical clustering of Euclidean distances. Each bar represents a sample from an individual bird

The majority of taxa from both eastern bluebirds and tree swallows were represented by seven bacterial phyla. The most common members of the microbiota were the Proteobacteria, Bacteroidetes, and Firmicutes (Fig. 2). In general, eastern bluebirds had a higher relative abundance of Bacteroidetes and Planctomycetes compared to the tree swallows. At the level of genus, eastern bluebird microbiotas had higher proportions of *Rhodospirillum*, *Bacteroides*, and *Parabacteroides*, while tree swallows had higher proportions of *Providencia* and “Candidatus Arthromitus*”* (Additional file 1: Fig. S1).

**Fig. 3:**
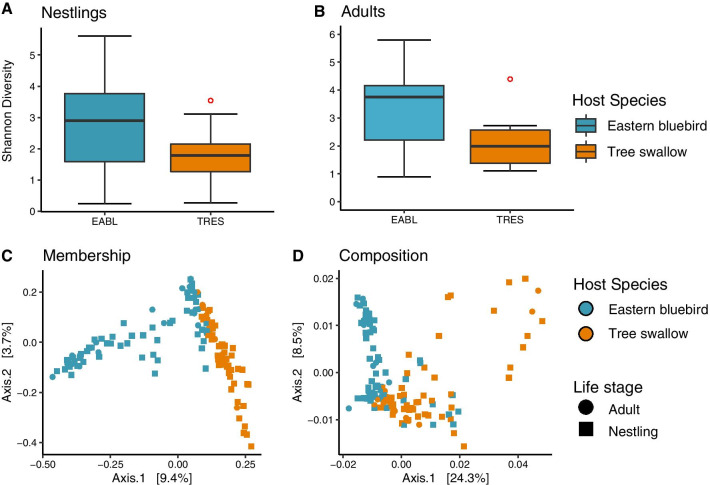
Shannon diversity boxplots for nestlings (**a**) and adult birds (**b**) of both species. Midline represents the median Shannon diversity value. Red points indicate outliers. Principal coordinates ordination of (**c**) unweighted (membership) and (**d**) weighted (composition) UniF rac distances for all birds. Each point represents an individual sample colored according to host species. Point shape indicates host life stage

The microbiotas of both nestling and adult eastern bluebirds had higher median Shannon diversity than those of tree swallows (Additional file 1: Table S1; Fig. 3a, b). Microbiotas of bluebirds and tree swallows were also significantly different in membership (PERMANOVA, F_2,134_ = 4.65, *p*_*adj*_ = 0.016) and composition (PERMANOVA, F_2,134_ = 8.59, *p*_*adj*_ = 0.016) (Fig. 3c, d). Host species identity explained more variation in membership (e.g. presence or absence of bacterial OTUs) than abundance-weighted composition (Additional file 1: Table S2). Because host species identity had a strong effect on microbiotas, we analyzed the effects of heat treatment and parasitism on bacterial beta diversity separately for each species. We also limited these analyses to only the microbiota of nestlings since microbiota varied significantly according to host life stage (Additional file 1: Table S2).

### Effects of temperature and nest parasitism on nestling microbiota

**Fig. 4:**
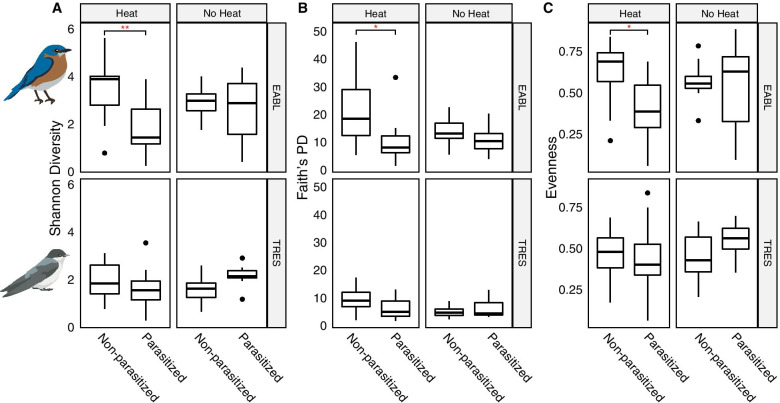
Effect of heat and parasite treatments on alpha diversity of eastern bluebird and tree swallow nestling microbiotas. Panels depict **a**) Shannon diversity, **b**) Faith’s phylogenetic diversity, and **c**) Evenness. Horizontal bars represent median values while solid points indicate outliers. Significance codes: ** = *p*_*adj*_ < 0.001, * = *p*_*adj*_ < 0.05

For alpha diversity, we found that parasitized eastern bluebirds had lower bacterial richness than non-parasitized birds, regardless of heat treatment or life stage. In particular, heat treated and parasitized nestlings had the lowest bacterial richness of any treatment condition in this species (Additional file 1: Table S1). In contrast, parasitized tree swallow nestlings had higher richness than non-parasitized nestlings, but only when heat was not applied. In the heat treated tree swallow nestlings, bacterial richness slightly decreased in the parasitized cohort (Additional file 1: Table S1). There were too few observations of adult tree swallows to make such a comparison in this cohort, but there was a trend toward lower bacterial richness in parasitized adults in the non-heat treated group (Additional file 1: Table S1). Heat and parasite treatments impacted the alpha diversity of bluebird and tree swallow microbiotas, but the significance of these effects varied between host species. For both species, we found that heat treatment alone did not significantly alter microbial richness, phylogenetic diversity, or community evenness (Fig. 4, Additional file 1: Table S1). Overall, heat treatment in combination with parasitism significantly reduced all three alpha diversity estimates in bluebirds, while tree swallows were not affected (Fig. 4).

We tested the effects of heat and parasite treatments on each species at both the community level (i.e., beta diversity) and at the level of individual bacterial ASV. We used PERMANOVA to test the effects of these treatments on beta diversity of whole bacterial communities. For the eastern bluebirds, we found that parasite treatment and heat treatment significantly impacted microbial beta diversity in both membership and composition, but the interaction between these variables only significantly impacted composition (Additional file 1: Table S3). In contrast, tree swallow microbiota membership and composition were not significantly impacted by parasitism or heat treatment, nor their interaction (Additional file 1: Table S3).

We next tested the effects of heat, parasite treatment, and their interaction on the individual abundances of 13 bacterial phyla. Models for several bacterial taxa failed to converge, likely due to a small number of observations. For the remaining taxa which achieved model convergence, we report parameter estimates and results of significance tests corrected for the false discovery rate (Additional file 1: Table S4). The abundances of Fusobacteria, Actinobacteria, Chloroflexi, and Synergistes were negatively associated with the presence of nest parasites in eastern bluebirds, while only Chloroflexi abundance was impacted by heat treatment alone. For tree swallows, we did not detect an effect of either heat or parasitism on the abundance of any of the tested phyla. For both nestling species, the interaction between heat and parasitism was not determined to have a significant effect on the abundance of any one bacterial phylum.

## Discussion

This study showed that experimental heat treatment and parasite manipulation differentially impacts the microbiotas of two avian hosts, eastern bluebirds and tree swallows. Host species identity explained most of the variation in microbiotas among the two bird species (Figs. 2 and 3, Additional file 1: Table S2), which is consistent with previous work showing strong patterns of bacterial host specificity in other wild vertebrates [35–38]. The bacterial communities of the two nestling species also showed idiosyncratic responses to both heat treatment and nest parasite treatment. Eastern bluebird nestling microbiotas showed a 14% decrease in mean richness in the presence of nest parasites when heat was not applied (Additional file 1: Table S1). When temperature was experimentally elevated, parasitized bluebird nestlings showed a 48% decrease in richness compared to the non-parasitized birds. On the other hand, tree swallow nestlings had similar microbiota alpha diversity in the presence or absence of nest parasites. When heat was applied, alpha diversity decreased by 22% in the parasitized nestlings compared to those in parasite-free nests, though this reduction was not statistically significant. Heat application alone did not significantly reduce alpha diversity in eastern bluebirds when parasites were absent, though there was a trend toward lower richness as well (Additional file 1: Table S3).

The results of the heat treatment experiment are broadly concordant with previous studies examining the effects of heat on vertebrate gut bacterial communities. In cattle and tadpoles, heat alone appears to have no effect on microbiome alpha diversity [39, 40]. In other cases, higher ambient temperatures were found to be associated with lower alpha diversity in the gut microbiota of lizards [41], laying chickens [42], and salamanders [43]. Because endothermic animals such as birds are able to maintain thermal homeostasis, it is not likely that ambient temperature directly affects their gut microbiotas. Rather, the shifts we observed likely result from changes in host physiology spurred by the higher temperatures (e.g., [44, 45]). Accumulating evidence suggests that passerine nestlings respond physiologically to changes in nest temperature; elevated temperatures are associated with decreased body mass and wing length and higher corticosterone levels [32, 46, 47]. The exact mechanisms by which these physiological changes might alter gut microbiota composition remain largely unknown. One hypothesis is that corticosterone alters the structure of the gut mucosal barrier, which exerts an effect on the microbiota. For example, elevated cortisol levels lead to reduced mucin production in urban squirrels [48]. Mucus may regulate the composition of the microbiota, and it is thought that perturbations to the mucus layer (e.g., by changes in host physiology in response to stress) may compromise the stability of these communities and give rise to dysbiosis [49]. Future studies could examine links between mucosal thickness, corticosterone levels, and microbiota attributes in birds experimentally exposed to heat to further develop our understanding of these interactions.

The two host species’ microbiotas also responded differently to the treatments in terms of beta diversity. Both experimental variables impacted the membership and composition of eastern bluebird nestling microbiotas, including the interaction of the heat and parasitism treatments (Additional file 1: Table S4). However, tree swallow beta diversity was robust to both experimental treatments, with neither heat, parasitism, nor their interaction, having a significant influence on either community membership or structure. These results are consistent with previous work on this system, which showed that while both species are tolerant to the presence of parasitic nest flies in terms of nestling mortality, tree swallows are both more tolerant *and* more resistant to the sublethal effects of the parasites than bluebirds [27, 30]. However, given that heat application still resulted in a trend toward lower alpha diversity in tree swallow nestlings, our results suggest that heat stress may interfere with the ability of both species to maintain stable associations with their microbiotas in the presence of nest parasites.

Interactions between temperature and parasitism have been reported in other systems, though few studies explicitly incorporate effects on host gut microbiota. In three-spined sticklebacks, rates of tapeworm infection were modulated by ambient temperature such that parasites were more easily able to exploit hosts in warmer experimental conditions [50]. *P. sialia* is an ectoparasite that feeds non-subcutaneously on nestlings, and thus may respond differently to heat than internal parasites, which are buffered from changes in the broader environment due to living within their hosts. Evidence suggests that elevated temperatures result in higher parasite burdens in eastern bluebird nests, while the opposite effect is observed for tree swallows [32]. Eastern bluebirds therefore appear to be more susceptible to the interaction of heat stress and parasitism than tree swallows, and our results suggest that these effects in combination have the power to alter their gut bacterial communities. Shifts in microbial composition may signal destabilization of the microbiota in ways that are maladaptive for their hosts. For example, urbanization-driven changes in the microbiotas of American white ibises were significantly associated with pathogen prevalence, suggesting that wild hosts may suffer decreased fitness as a result of microbiome perturbation [51]. However, not all microbiome shifts are necessarily maladaptive. Future work using metagenomic or metatranscriptomic techniques could assess the functional implications of these shifts for hosts and determine the fitness consequences of altered microbial communities.

Heat and parasitism did not significantly impact the abundance of any bacterial phylum in tree swallow nestlings (Additional file 1: Table S4). In eastern bluebirds, we found that parasite presence caused a decrease in the abundance of Chloroflexi, Fusobacteria, Synergistes, and Actinobacteria, while heat treatment only had a significant effect on Chloroflexi abundance (Additional file 1: Table S4). The reduced abundance of these bacterial phyla has been linked to parasitism and disease outcomes in other study systems. One study found that treatment-induced reduction of Fusobacteria in tadpole microbiotas was linked to higher parasite susceptibility in adult frogs [33]. Similarly, IgY and IgG antibody production has been strongly correlated with Chloroflexi and Fusobacteria abundance in wild Galapagos mockingbirds [29] and a mouse model [52], respectively, suggesting that these bacterial taxa may be instrumental for priming the immune system in early life. Phylum Synergistes is a recently described bacterial phylum that is common in animal guts but about which relatively little is known. However, at least one study comparing captive and wild wood grouse (*Tetrao urogallus)* found that Synergistes was completely lost and Actinobacteria significantly reduced in captive birds and suggested that these changes may compromise the performance of the cecum in birds released into the wild [53].

Taken together, these results suggest that parasitized eastern bluebird nestlings are sensitive to exposure to heat and parasitism. Parasitism may reduce the abundance of key microbiota members that are known to prime immune function, and as a result, the birds may be more susceptible to heat stress and other environmental stressors throughout their lives [32]. The interaction of temperature and parasitism on host-associated microbiotas may be of particular relevance in the context of climate change. Mean global surface air temperature is projected to increase by 1.4°–5.8 °C by 2100 relative to 1990 [54], which falls within the range of our experimentally elevated nest temperatures relative to normal [55]. Given our results, we might expect eastern bluebirds to fare worse under climate change than tree swallows. An interesting mechanistic hypothesis for this prediction is that the tree swallow microbiotas may be mediating their susceptibility to both heat and parasitism. Previous studies have demonstrated that tree swallows mount immune responses to *P. sialia* that reduce parasite burdens [27], and given the links between the microbiota and immune function, we speculate that the ability of tree swallows to maintain stable associations with their microbes may partially be explained by their immune performance. In general, internal microbiota diversity is correlated with immune complexity across all organisms, suggesting a role for the immune system in promoting stable associations with microbes [56]. Eastern bluebirds, on the other hand, do not produce elevated immune responses to parasites and sustain twice the parasite burden per gram of nestling compared with tree swallows [27]. In contrast with tree swallows, eastern bluebird microbiotas shifted in response to heat and parasitism, suggesting that their immune systems and microbiotas may be less coupled. Future studies elucidating these mechanisms would add depth to our understanding of the importance of the microbiome in mediating host immune performance.

Given that both parasitism and heat reduced the abundance of key microbiota members associated with immunity, we predict that the net effects of a warming world and natural nest parasitism may threaten eastern bluebird survival in the future. This prediction is consistent with other studies raising concerns about the impacts of climate change on bluebird reproduction and survival (e.g., [57, 58]). In contrast, a long-term study on breeding performance found that between the periods 1962–1972 and 2006–2016, tree swallow reproductive performance increased as a result of earlier breeding induced by warmer winter temperatures [59]. Thus, the combined effects of a warming climate in concert with variation in physiological responses to heat and nest parasites are likely to impact eastern bluebirds and tree swallows differently. Future work should explicitly test the survival and lifetime fitness costs of nestlings exposed to heat in early life and incorporate metatranscriptomic assays or metabolomics to assess the functional contributions of gut bacteria in determining host immune outcomes.

## Conclusions

In conclusion, this study demonstrated that two species of free-ranging avian hosts respond differently to heat and parasitism at the level of the microbiota, and that specific bacterial phyla change in relative abundance in response to the treatments. Our work joins a growing body of literature suggesting that the gut microbiota may have the power to mediate external stressors such as heat and ectoparasite presence in wild hosts. This experimental study and future work will be instrumental in predicting how natural host-parasite-microbe systems respond to a warming world.

## METHODS

### Study Site

The experiment was conducted at the University of Minnesota Itasca Biological Station and Laboratories (47°13′33″N, − 95°11′42″W), Minnesota, USA from May to August 2018. In 2018, approximately 170 nest boxes were established on private properties and near Itasca State Park. Tree swallows and eastern bluebirds are abundant in the area and nest readily in artificial cavities. At this site, *Protocalliphora sialia* is the main species of blowfly that parasitizes both bluebird and tree swallow nests [27]. Tree swallow clutch size ranges from one to nine eggs incubated for 13–14 days. Nestlings spend an average of 20 days in the nest [27]. Bluebird clutch size ranges from one to seven eggs, which are incubated for 13–14 days. Nestlings spend an average of 18.8 days in the nest [60].

### Experimental manipulation of nest parasites and temperature

We checked nest boxes once per week for evidence of nest construction. Once eggs were found, we recorded lay date and checked nests every other day until the eggs hatched. During the nestling stage, we conducted a fully factorial experiment by manipulating parasite presence (parasites vs. no parasites) and nest temperature (heat treatment vs. no heat) for both eastern bluebird and tree swallow nests (Fig. 1). At hatching, the nestlings and the top liner of the nests were removed for parasite manipulation. For the control treatment, we sham treated nests with sterile water to allow for natural parasitism (parasitized). For the experimental treatment, we treated nests with a 1% permethrin solution to remove all parasites (non-parasitized; Permectrin II) [29, 30]. We initially determined parasite treatment for each species by a coin flip, and alternately assigned all subsequent nests to a treatment.

For the heat treatment, we used a metal spatula to lift nest material from the bottom of the box and placed a fresh UniHeat 72 + Hour heat pack (heat-treated) or an exhausted heat pack (non-heat treated) in the open space. The packs contain a mixture of charcoal, iron powder, vermiculite, salt, sawdust, and moisture, and produce elevated temperatures between 35 and 40 °C for two days when exposed to the air [61]. Nest boxes were revisited every two days to replace active heat packs so that nest boxes were maintained at constant elevated temperature until nestlings were 10 days old. For control nests, nest material was lifted with a metal spatula to control for nest disturbance. Heat packs were always checked for parasites before they were removed; any parasites that were on the heat pack were returned to the nest. Heat treatment for each species was initially determined by a coin flip and all subsequent nests were assigned to alternating treatments. Mean daily temperature for heated nests was 31.1 °C (± 5.47) and 26.0 °C (± 3.66) for non-heated nests, and throughout the course of the experiment, fluctuations in nest temperature were similar for both treatments (Additional file 1: Fig. S2).

When nestlings were approximately 6 days old, we captured adult mothers on the nest to sample their microbiotas. To catch adults, we placed a transparent film over the opening to the nest box that allowed birds to enter, but not to exit. Once an adult entered the box, we retrieved them and then removed the film from the opening. After capturing, adults were placed in a flat-bottomed paper bag containing a vinyl-coated hardware cloth fencing and sterile tray for 3–5 min until defecation [62]. The feces were removed from the tray to a sterile tube with approximately 500 µL of DNA/RNA Shield (Zymo Research, Inc.), placed on ice in the field for up to 6 h, and then stored in a − 80 °C freezer until bacterial DNA was extracted.

When nestlings were approximately 10 days old, we collected feces from them and ended the heat treatment. To collect feces, nestlings were removed from the nest and held over a sterile weigh-boat until they defecated. The fecal sample was then moved from the tray to a sterile tube with approximately 500µL of DNA/RNA Shield (Zymo Research, Inc.), placed on ice in the field for up to 6 h, and then stored in a -80 °C freezer until the bacterial DNA was extracted. The samples were then transported to the University of Connecticut and stored in a -80 °C freezer for downstream 16S sequencing. Although studies show that the bacterial community in avian feces does not always represent the entire digesta of the host (e.g., in the cecum; [63]), fecal samples are generally representative of the bacterial community in the large intestines [63, 64] and are used when hosts cannot be euthanized [65].

When nests were empty, they were carefully removed from the nest box and stored in a gallon-sized, labeled plastic bag. To confirm that the sham-treated nests were indeed parasitized naturally, nest material was then dissected over trays lined with a white piece of paper. All *P. sialia* larvae (1st, 2nd, and 3rd instars), pupae, and pupal cases were counted to determine total parasite abundance for each nest for Albert et al. [32].

### DNA isolation and 16S rRNA gene sequencing

Before starting the extraction, samples were centrifuged for 10 min at 10,000 rpm and 4 °C and the supernatant (i.e. DNA/RNA Shield) was then removed. Total DNA was extracted from nestling feces using a Qiagen PowerFecal DNA Isolation Kit (QIAGEN, Inc.). DNA extractions were then sent to the University of Connecticut Microbial Analysis, Resources and Services for sequencing with an Illumina MiSeq platform and v2 2 × 250 base pair kit (Illumina, Inc.). We also sequenced a DNA blank using sterile water as input. Although the negative blank concentration was too low to be quantified (< 0.05 ng/µL), we still subjected this sample to the same PCR procedure and sequencing as our samples of interest. Bacterial libraries were constructed by amplifying the V4 region of the 16S rRNA gene using primers 515F and 806R [66] and with Illumina adapters and dual indices [67]. Samples were amplified in triplicate 15 μl reactions using Go-Taq DNA polymerase (Promega) with the addition of 3.3 μg BSA (New England BioLabs). To overcome inhibition from host DNA, 0.1 pmol primer without the indexes or adapters was added to the mastermix. The PCR reaction was incubated at 95 °C for 3.5 min, the 30 cycles of 30 s at 95.0 °C, 30 s at 50.0 °C and 90 s at 72.0 °C, followed by final extension at 72.0 °C for 10 min. PCR products were pooled for quantification and visualization using the QIAxcel DNA Fast Analysis (Qiagen). PCR products were normalized based on the concentration of DNA from 250–400 bp then pooled using the epMotion 3075 liquid handling robot. The pooled PCR products were cleaned using Omega Bio-Tek Mag-Bind Beads according to the manufacturer’s protocol using 0.8 × beads to PCR product volume. The cleaned pool was sequenced on the MiSeq using v2 2 × 250 base pair kit (Illumina, Inc).

### Bioinformatic and statistical analyses

A total of 138 fecal samples were successfully sequenced. Raw forward and reverse reads for each sample were imported into QIIME2 v. 2020.8 [68]. Initial quality control was performed manually by summarizing demultiplexed sequences and visualizing per-base quality scores in QIIME2. Next, we used the DADA2 algorithm to trim low-quality base calls (as identified in the previous summary step), join forward and reverse reads, and identify and remove chimeric sequences [69]. Of a total of 7,516,369 raw reads, 6,489,278 passed the DADA2 denoising step (86%). The average number of merged, non-chimeric reads was 45,673 across all samples (SE_Mean_ ± 3,918). The total number of ASVs recovered after contaminant filtering was 8107. The resulting feature table of amplicon sequence variants (ASVs) was then used to construct a bacterial 16S phylogeny, using the MAFFT alignment algorithm (q2-alignment) and the FastTree maximum likelihood estimation (q2-phylogeny) plugin [70, 71]. Bacterial taxonomy was assigned using the q2-feature-classifier [72] naive Bayesian classifier against the Greengenes 13_8_99% OTUs reference database [73]. After visual inspection of the resulting taxonomic bar plots, we filtered out mitochondrial and chloroplast sequences from the ASV feature table and exported the data for further analysis in the R statistical environment [74].

Basic data preprocessing was performed using packages *phyloseq* v. 1.32.0 [75] and *microbiome* v. 2.1.25, and *decontam* v. 1.8.0 [76, 77]. Analysis of contaminants with *decontam* identified only a single ASV as a potential contaminant, so we removed this ASV from the feature table prior to all statistical analyses. First, we filtered the feature table to retain only those samples with at least 1000 total reads, which reduced the dataset to 135 samples. Because microbiome samples are prone to ambient laboratory contamination [78, 79], we also used *decontam* v. 1.8.0 [77] to filter out potential contaminant ASVs from the samples based on the prevalence of bacteria in the extraction negative control. We performed alpha diversity calculations on the contaminant-filtered dataset, computing Shannon diversity for each sample. Prior to beta diversity analyses, we performed a Hellinger transformation (i.e., square root of the relative abundance given at the scale [0,1]) on the ASV table to account for differences in library size [80].

Because our dataset contained relatively few adult mothers (*n* = 29), we decided to focus on only nestlings for the beta diversity analyses (*n = 107*). We used R package *vegan* [81] to compute alpha (Shannon, Faith’s PD, Evenness) and beta (Bray–Curtis, UniF rac) diversity metrics for all samples [82]. To test for differences in alpha diversity among experimental treatments, we compared treatments within species using the Wilcoxon test and applying a Bonferroni correction to all significance values. To compare microbiota among experimental conditions, we conducted PERMANOVA tests on both unweighted (composition) and weighted (structure) UniF rac distances. Distance matrices were estimated separately for each bird species. The full model call was (dist.matrix ~ Parasite + Heat + Parasite * Heat). An important assumption of PERMANOVA is homogeneity of dispersion among groups, so we also performed a beta dispersion test for each distance matrix using the command “betadisper” in *vegan*. Because we tested the same hypotheses separately on each bird species, we controlled for multiple testing by adjusting all *p*-values using a Benjamini–Hochberg correction.

To specifically test for the effects of nest temperature treatment and parasite treatment on detection of gut microbial taxa, we used general linear models with mixed effects (GLMMs). To reduce the overall number of ASVs tested, we pruned the dataset to remove those ASVs that were not detected in at least 10 individual birds as well as those that had fewer than 100 total read counts. This data stringency left us with a total of 16 phyla of 112 bacterial families available for testing. Using package *glmmTMB* [83], we constructed models testing the effects of parasite treatment, heat treatment, and the interaction between parasitism and heat on each bacterial phylum. We fit models using a type-2 negative binomial distribution with a log link and modeled nest ID as a random effect to account for brood sampled from the same nests. Because microbiome features tables include many zeroes, we additionally included a zero-inflation component modelled on a single intercept. All *p*-values were adjusted for false discovery rate with a Benjamini–Hochberg correction.

All visualizations were created using *phyloseq* [75] and *ggplot2* [84]. Bar plots were created using *fantaxtic* (https://github.com/gmteunisse/Fantaxtic) and all color palettes are adapted from *wesanderson* (https://github.com/karthik/wesanderson).

## Supplementary Information

**Additional file 1: Fig. S1**. Microbiotas at genus level.** Fig. S2**. Mean daily temperature range.** Table S1**. Alpha diversity table.** Table S2**. Overall beta diversity table.** Table S3**. Beta diversity modeled separately by host species.** Table S4**. Mixed effects model results.

## Data Availability

All statistical analyses and code can be accessed publicly at https://github.com/MelissaIngala/ItascaBirdMicrobiota. Raw 16S rRNA data is publicly archived on the NCBI SRA under BioProject # PRJNA733473.
