# Supplementary material for: Differential effects of elevated nest temperature and parasitism on the gut microbiota of wild avian hosts
Source: Anim Microbiome. 2021 Oct 2;3:67. doi: 10.1186/s42523-021-00130-3 (PMC8487522; doi:10.1186/s42523-021-00130-3)
Supplement: Supplementary file 1 — Additional file 1: Fig. S1. Microbiotas at genus level. Fig. S2. Mean daily temperature range. Table S1. Alpha diversity table. Table S2. Overall beta diversity table. Table S3. Beta diversity modeled separately by host species. Table S4. Mixed effects model results. [file 42523_2021_130_MOESM1_ESM.docx]

**Supplemental Information**


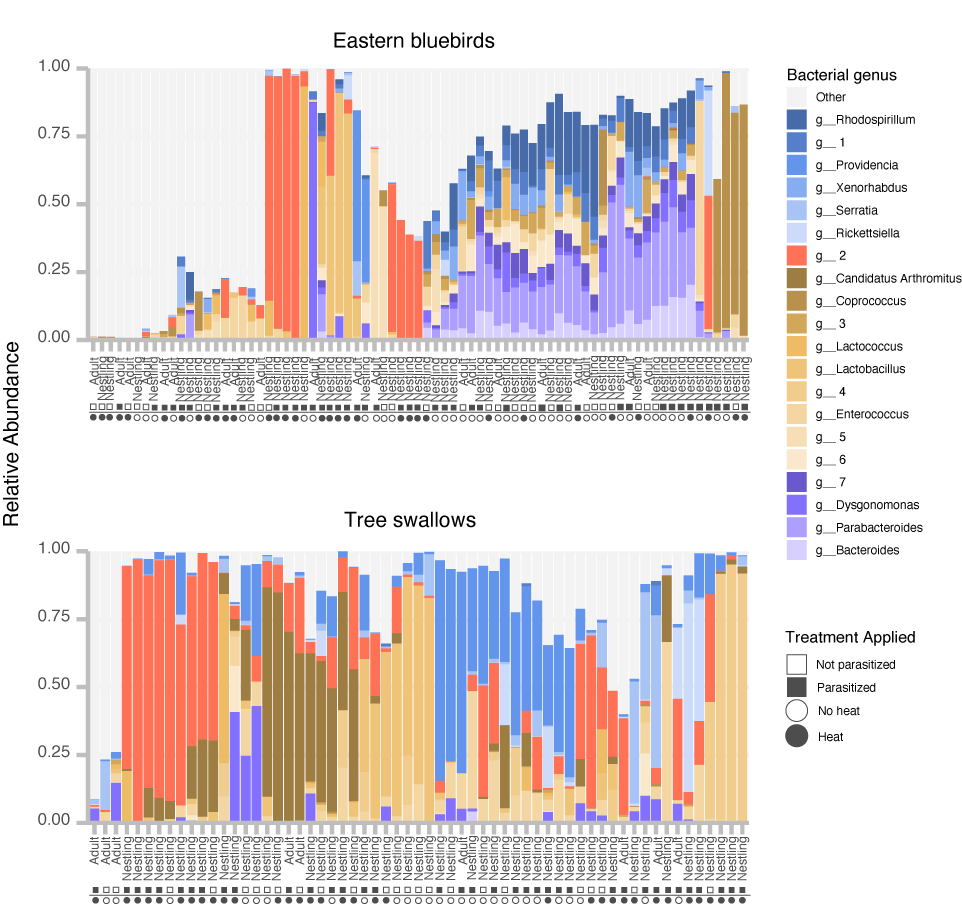


**Supplemental Figure S1**. Proportional abundance of bacteria in the microbiotas of eastern bluebirds and tree swallows summarized at the level of genus. Samples are organized by hierarchical clustering of Euclidean distances. Unidentified genera are numbered sequentially. The top 20 genera are shown, and all others are collapsed into the “other” category.


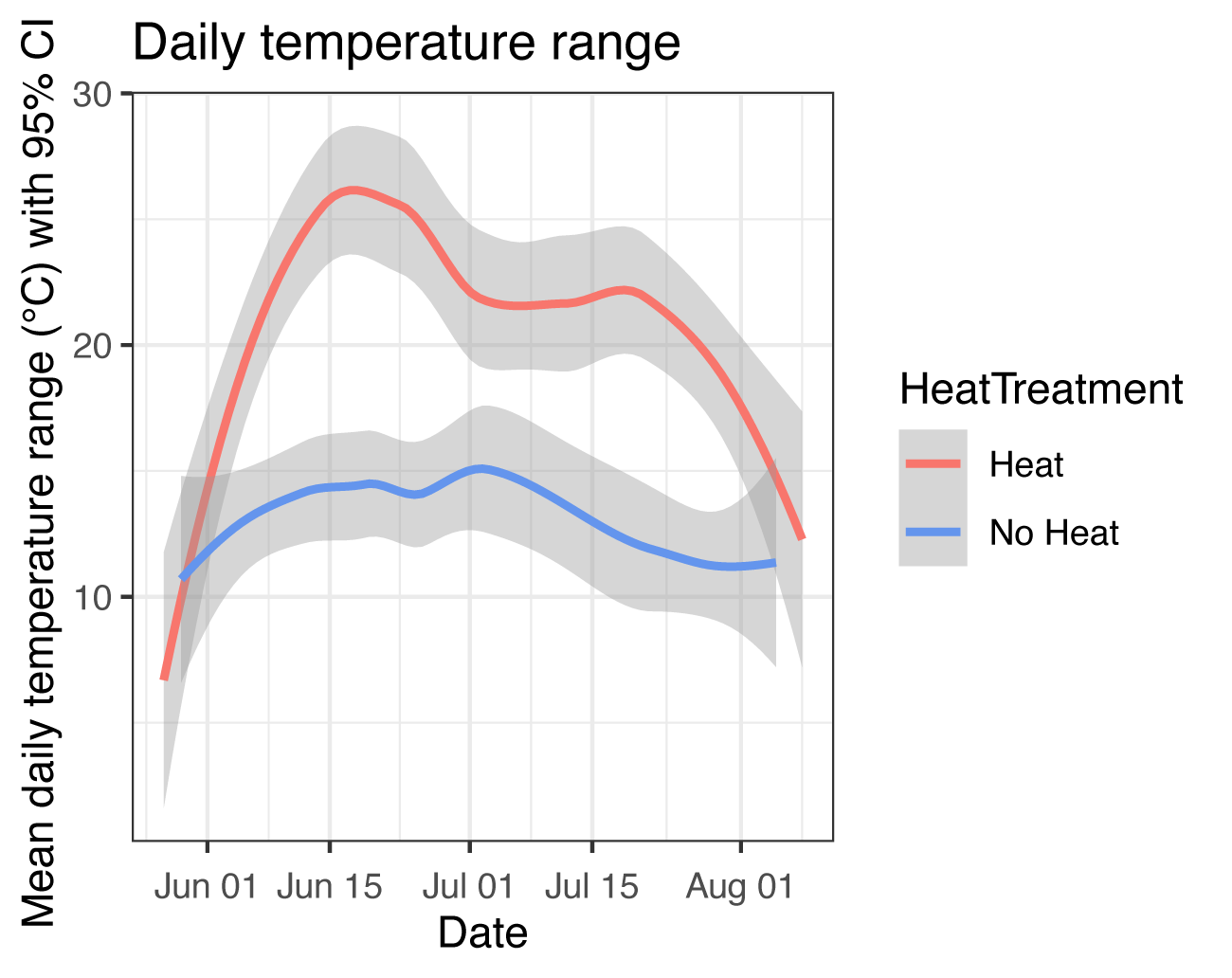


**Supplemental Figure S2.** Mean daily temperature range throughout the course of the experiment. Grey shadows represent 95% CI.

**Supplemental Table S1.** Effect of experimental heat and parasite treatment on alpha diversity of bird microbiotas. Mean Shannon diversity is reported ± standard error. Numbers in parentheses indicate sample size for each condition.

|  | **No heat** | |  | **Heat** | |
| --- | --- | --- | --- | --- | --- |
|  | **Non-parasitized** | **Parasitized** |  | **Non-parasitized** | **Parasitized** |
| **Eastern bluebirds** |  |  |  |  |  |
| Nestlings | 2.90 ± 0.2 (10) | 2.55 ± 0.33 (16) |  | 3.49 ± 0.33 (14) | 1.82 ± 0.28 (15) |
| Adults | 3.71 ± 0.33 (6) | 2.88 ± 0.46 (6) |  | 4.99 ± 0.81 (2) | 2.89 ± 0.56 (6) |
|  |  |  |  |  |  |
| **Tree swallows** |  |  |  |  |  |
| Nestlings | 1.58 ± 0.15 (13) | 2.16 ± 0.14 (10) |  | 1.99 ± 0.28 (9) | 1.56 ± 0.16 (20) |
| Adults | 3.56 ± 0.83 (2) | 1.96 ± 0.60 (2) |  | 1.38 (1) | 1.76 ± 0.23 (4) |

**Supplemental Table S2.** PERMANOVA results for Bray-Curtis distance matrix and both membership (unweighted UniFrac) and composition (weighted UniFrac) for all birds.

|  | **Bray-Curtis** | | **Unweighted UniF** **rac** | | **Weighted UniF** **rac** | |
| --- | --- | --- | --- | --- | --- | --- |
|  | r^2^ | *P*_adj_ | r^2^ | *P*_adj_ | r^2^ | *P*_adj_ |
| **Host species** | 0.11142 | 0.012* | 0.11144 | 0.012* | 0.06445 | 0.016* |
| **Parasite Treatment** | 0.00859 | 0.012* | 0.00876 | 0.012* | 0.00850 | 0.016* |
| **Heat Treatment** | 0.01851 | 0.012* | 0.02080 | 0.012* | 0.01067 | 0.016* |
| **Life Stage** | 0.01154 | 0.012* | 0.00973 | 0.012* | 0.00901 | 0.016* |

*Significant at (*P*_adj_ < 0.05)

**Table S3.** Effect of parasite treatment, heat treatment, and their interaction on beta diversity of eastern bluebird and tree swallow microbiotas. Values labeled * are significant at *P_adj_* ≤ 0.05.

|  | **Unweighted UniF** **rac**  **(Membership)** | | **Weighted UniF** **rac**  **(Composition)** | |
| --- | --- | --- | --- | --- |
|  | **r^2^** | ***p*_adj_** | **r^2^** | ***p*_adj_** |
| **Eastern Bluebirds** |  |  |  |  |
| Parasite Treatment | 0.024 | 0.047 * | 0.029 | 0.047 * |
| Heat Treatment | 0.027 | 0.047 * | 0.041 | 0.018 * |
| Parasite*Heat | 0.018 | 0.4370 | 0.029 | 0.047 * |
| **Tree Swallows** | | | | |
| Parasite Treatment | 0.019 | 0.80 | 0.019 | 0.80 |
| Heat Treatment | 0.023 | 0.46 | 0.036 | 0.33 |
| Parasite*Heat | 0.025 | 0.276 | 0.025 | 0.71 |

**Table S4.** Mixed effects model results (coefficient and standard deviation) for the impacts of heat and parasite manipulation on 16 bacterial phyla, split amongst eastern bluebirds and tree swallows nestlings. Values shown in bold are significant at *p_adj_* < 0.05 after Benjamini-Hochberg correction. “NA” values indicate that the model failed to converge for the specified bacterial taxon. W = parasitized condition, NH = no heat applied

| **Host Species** | **Bacterial Phylum** | **Intercept** | **Parasitism (Parasitized)** | **Heat (No Heat)** | **Parasitism*Heat** |
| --- | --- | --- | --- | --- | --- |
| **Eastern Bluebirds** | Tenericutes | **6.51 (± 1.75)** | 3.35 (± 2.23) | -1.23 (± 2.57) | -5.94 (± 3.4) |
|  | Proteobacteria | **9.88 (± 0.34)** | -0.75 (± 0.48) | 0.70 (± 0.53) | 0.79 (± 0.70) |
|  | Fusobacteria | **6.32 (± 1.53)** | **-4.99 (± 2.13)** | -32.6 (± 158411) | 31.1(± 158411) |
|  | Deferribacteres | **3.19 (± 0.89)** | -0.62 (± 1.24) | 0.80 (± 1.38) | -0.25 (± 1.83) |
|  | Crenarchaeota | **3.88 (± 0.89)** | -0.26  (± 0.0002) | -1.08 (± 1.38) | -5.56 (±  0.00004) |
|  | Verrucomicrobia | **5.67 (± 0.59)** | -0.81 (± 0.82) | -0.61 (± 0.91) | 1.27 (± 1.21) |
|  | Actinobacteria | **6.53 (± 0.48)** | **-1.38 (± 0.63)** | -0.0009 (± 0.72) | 0.33  (± 0.92) |
|  | Chlamydiae | 3.92 (± 2.23) | -0.63 (± 3.10) | -4.27 (± 3.48) | 8.33 (± 4.60) |
|  | Acidobacteria | -5.26 (± 4.72) | -2.52 (± 4.26) | -20.0 (± 15342) | 19.40  (± 15342) |
|  | Chloroflexi | **4.93 (± 1.0)** | **-5.24 ± (1.43)** | **-3.21 ± (1.56)** | 2.54 (± 2.12) |
|  | p__Unknown | NA | NA | NA | NA |
|  | Synergistetes | **4.91 (± 1.33)** | **-5.13 (± 1.87)** | 0.42 (± 2.05) | 1.37 (± 2.74) |
|  | Bacteroidetes | **9.14 (± 1.28)** | -1.53 (± 1.17) | 0.24 (± 1.44) | 1.25 (± 1.65) |
|  | Firmicutes | **9.24 (±0.50)** | -0.37 (± 0.68) | 0.23 (± 0.78) | 0.018 (±1.01) |
|  | Cyanobacteria | NA | NA | NA | NA |
|  | Planctomycetes | **4.16 (± 0.95)** | -2.68 (± 1.33) | -1.78 (± 1.48) | 2.34 (± 1.96) |
| **Tree Swallows** | Tenericutes | **8.87 (±0.55)** | 0.31 (±0.67) | -0.42 (±0.72) | -1.13 (±0.97) |
|  | Proteobacteria | **9.65 (± 0.62)** | -0.46 (±0.82) | -0.63 (±0.82) | 1.13 (±1.16) |
|  | Fusobacteria | NA | NA | NA | NA |
|  | Deferribacteres | -24.2 (± 39533) | -0.93 (± 60975) | -0.97 (± 69930) | 3.87 ( ± 84132) |
|  | Crenarchaeota | NA | NA | NA | NA |
|  | Verrucomicrobia | NA | NA | NA | NA |
|  | Actinobacteria | **5.46 (± 0.66)** | -0.70 (± 0.80) | -1.53 (± 0.86) | 0.94 (± 1.16) |
|  | Chlamydiae | NA | NA | NA | NA |
|  | Acidobacteria | -24.16 (± 39533) | -0.93 (± 60975) | -0.97 (± 69930) | 3.87 (± 84132) |
|  | Chloroflexi | 24.2 (± 39533) | -0.93 (± 60975) | -0.97 (± 69930) | 3.87 (± 84132) |
|  | p__Unknown | -24.16 (± 39533) | -0.93 (± 60975) | -0.97 (± 69930) | 3.87 (± 84132) |
|  | Synergistetes | NA | NA | NA | NA |
|  | Bacteroidetes | **7.55 (± 0.96)** | -0.38 (± 1.15) | 0.99 (± 1.25) | -1.85 (±1.67) |
|  | Firmicutes | **9.62 (± 0.41)** | -0.35 (± 0.50) | -0.30 (± 0.54) | 0.15 (± 0.72) |
|  | Cyanobacteria | 2.87 (± 2.54) | -1.12 (± 3.06) | -0.62 (± 3.30) | 0.072 (± 4.44) |
|  | Planctomycetes | -24.16 (± 39533) | -0.93 (± 60975) | -0.97 (± 69930) | 3.87 (± 84132) |
